# Effects of ENSO and Temporal Rainfall Variation on the Dynamics of Successional Communities in Old-Field Succession of a Tropical Dry Forest

**DOI:** 10.1371/journal.pone.0082040

**Published:** 2013-12-12

**Authors:** Susana Maza-Villalobos, Lourens Poorter, Miguel Martínez-Ramos

**Affiliations:** 1 Escuela Nacional de Estudios Superiores, Unidad Morelia, Universidad Nacional Autónoma de México, Morelia, Michoacán, México; 2 Wageningen University, Forest Ecology & Forest Management Group, Wageningen, The Netherlands; 3 Centro de Investigaciones en Ecosistemas, Universidad Nacional Autónoma de México, Morelia, Michoacán, México; University of Saskatchewan, Canada

## Abstract

The effects of temporal variation of rainfall on secondary succession of tropical dry ecosystems are poorly understood. We studied effects of inter-seasonal and inter-year rainfall variation on the dynamics of regenerative successional communities of a tropical dry forest in Mexico. We emphasized the effects caused by the severe El Niño Southern Oscillation (ENSO) occurred in 2005. We established permanent plots in sites representing a chronosequence of Pasture (abandoned pastures, 0–1 years fallow age), Early (3–5), Intermediate (8–12), and Old-Growth Forest categories (n = 3 per category). In total, 8210 shrubs and trees 10 to 100-cm height were identified, measured, and monitored over four years. Rates of plant recruitment, growth and mortality, and gain and loss of species were quantified per season (dry *vs*. rainy), year, and successional category, considering whole communities and separating seedlings from sprouts and shrubs from trees. Community rates changed with rainfall variation without almost any effect of successional stage. Mortality and species loss rates peaked during the ENSO year and the following year; however, after two rainy years mortality peaked in the rainy season. Such changes could result from the severe drought in the ENSO year, and of the outbreak of biotic agents during the following rainy years. Growth, recruitment and species gain rates were higher in the rainy season but they were significantly reduced after the ENSO year. Seedlings exhibited higher recruitment and mortality rate than sprouts, and shrubs showed higher recruitment than trees. ENSO strongly impacted both the dynamics and trajectory of succession, creating transient fluctuations in the abundance and species richness of the communities. Overall, there was a net decline in plant and species density in most successional stages along the years. Therefore, strong drought events have critical consequences for regeneration dynamics, delaying the successional process and modifying the resilience of these systems.

## Introduction

Most theoretical and empirical studies on forest succession have focused on the role of light availability on the dynamics of tree communities [1], [2], [3]. By contrast, few studies have addressed the role of water availability in forest succession [4], especially in ecosystems that are severely limited by water, such as seasonal tropical dry forest (TDF), where mean annual rainfall is less than 1000 mm and the dry season spans several months with little or no rain at all. TDF represents a major biome in the world [5] and several functional, demographic, community, and ecosystem attributes of TDF plants are tightly matched to water availability as a fundamental resource [6], [7], [8].

Availability of water in TDF varies widely within seasons, among seasons, and among years [9], [10]. Current scenarios of global climate change predict that severe drought events, such as those caused by the El Niño Southern Oscillation (ENSO), will increase both in intensity and frequency in tropical regions [11], [12]. Under such climatic scenarios it is critical to understand to what extent temporal rainfall variability affects the natural regeneration and succession of TDF ecosystems in abandoned agricultural fields, which have become nowadays dominant components of tropical and non-tropical landscapes [13], [14].

Temporal variation in rainfall has been shown to have important effects on vital rates of woody TDF plants at their early life-cycle stages, especially when strong drought episodes occur [15], [16], [17], [18]. While recruitment and growth have been mostly linked with the rainy season, mortality has been observed to occur mostly during the dry season [16], [19], [20], [21], [22]. We can also expect that rates of gain and loss of species parallel these seasonal changes in vital rates, as variation in species number tends to be correlated with changes in abundance [23]. However, more studies are needed to verify the strength of such linkages, especially in successional environments in TDF.

During secondary succession, seedlings and sprouts of shrub and tree species may respond in different ways to changes in water availability. Sprouting has been proposed to be a major regeneration mechanism in TDF where strong water shortages may limit survival and growth of seedlings [24], [25], [26], whereas sprouts are less sensitive. Overall, survival and growth rates tend to be lower for seedlings than for sprouts [27], [28]. Likewise, in ecosystems outside the TDF, shrubs have shown to have higher recruitment, growth, and or survival rates than trees under the harsh conditions of early successional environments [29], [30]. Therefore, we hypothesize that the survival, growth and or recruitment of sprouts and shrubs would be higher than that of seedlings and trees at early successional stages and that such differences would reduce as succession advances.

Although it has been proposed that temporal variation in rainfall may affect the long-term dynamics of tree communities in TDFs [31], [32], little is known about the temporal signature of such effects [33]. The amount of precipitation within a given season is expected to have an immediate effect on plant survival because a severe and prolonged drought may cause the xylem vessels to cavitate leading to desiccation and eventual death of the impacted plants [34]. In contrast, plant growth and recruitment would vary not only with the amount of rain falling in a given season but also with the rainfall of previous seasons, which could modulate the storage of photosynthates needed to trigger future growth, reproduction. Stored carbohydrates could also allow plants to respond to, or recover from damage caused by herbivores and pathogens [25], [35], [36], [37], [38], [39], [40]. Rainfall variability may have a stronger effect on early than on late successional sites because in these early-successional sites environmental conditions are more severe and restrictive (i.e., high radiation and temperature levels, [8]) for the establishment of seedlings. In general, there is, however, a lack of knowledge about the immediate and long-lasting effects caused by rainfall regimes on the regeneration and successional dynamics of TDF.

In this paper we studied the effects of temporal variation in rainfall (within and among years) on the dynamics (*i.e*., gains and losses of plants and species) of regenerating communities of shrubs and trees (plants 10–100 cm height) over the early old-field succession of a TDF in western Mexico. In a previous paper [41] we analysed successional patterns of these communities, which strongly suggested that temporal variation in rainfall plays a critical role in such dynamics. In that paper we showed that the observed patterns had no relation at all with understory light availability or with structural variables of the established woody communities (trees and shrubs with DBH ≥1 cm). In this paper, we explicitly evaluate the importance of inter-annual and inter-seasonal rainfall events over a four-year period (2004–2007) that included a strong ENSO event. The evaluation was conducted considering the whole community, separating plant growth forms (shrubs *vs*. trees) and separating plant regeneration strategies (seedlings *vs*. sprouts). Specifically, we tested the following hypotheses: i) mortality and species loss rates are higher in dry seasons and drought years while recruitment, growth, and species gain rates are higher in rainy seasons and rainy years, ii) trees and seedlings are more prone than shrubs and sprouts to mortality during drought events, iii) changes in the plant community depend on the antecedent and current rainfall events, and iv) temporal variability in rainfall has stronger effects on community dynamics of early successional stages compared to late successional stages.

During the second year of our study period (in 2005), an ENSO event occurred, resulting in the lowest rainfall level observed in 30 years. This provided a unique opportunity to assess the effects of an extreme ENSO event on regenerating plant communities. Strictly speaking, this ENSO effect was not replicated, which makes it difficult to distinguish cause and effect. Yet, because we monitored the dynamics of regenerative communities in- before- and after the ENSO year, we are confident that the observed differences were mostly due to the extreme climate conditions.

## Materials and Methods

### Ethics Statement

Field activities into the Chamela Biological Field Station were conducted under the full permission of the Chamela Biological Field Station, Institute of Biology, National Autonomous University of Mexico. The rest of the field activities were conducted in leased sites for this study and did not require a specific permission from authorities.

### Study Site

**Figure 1:**
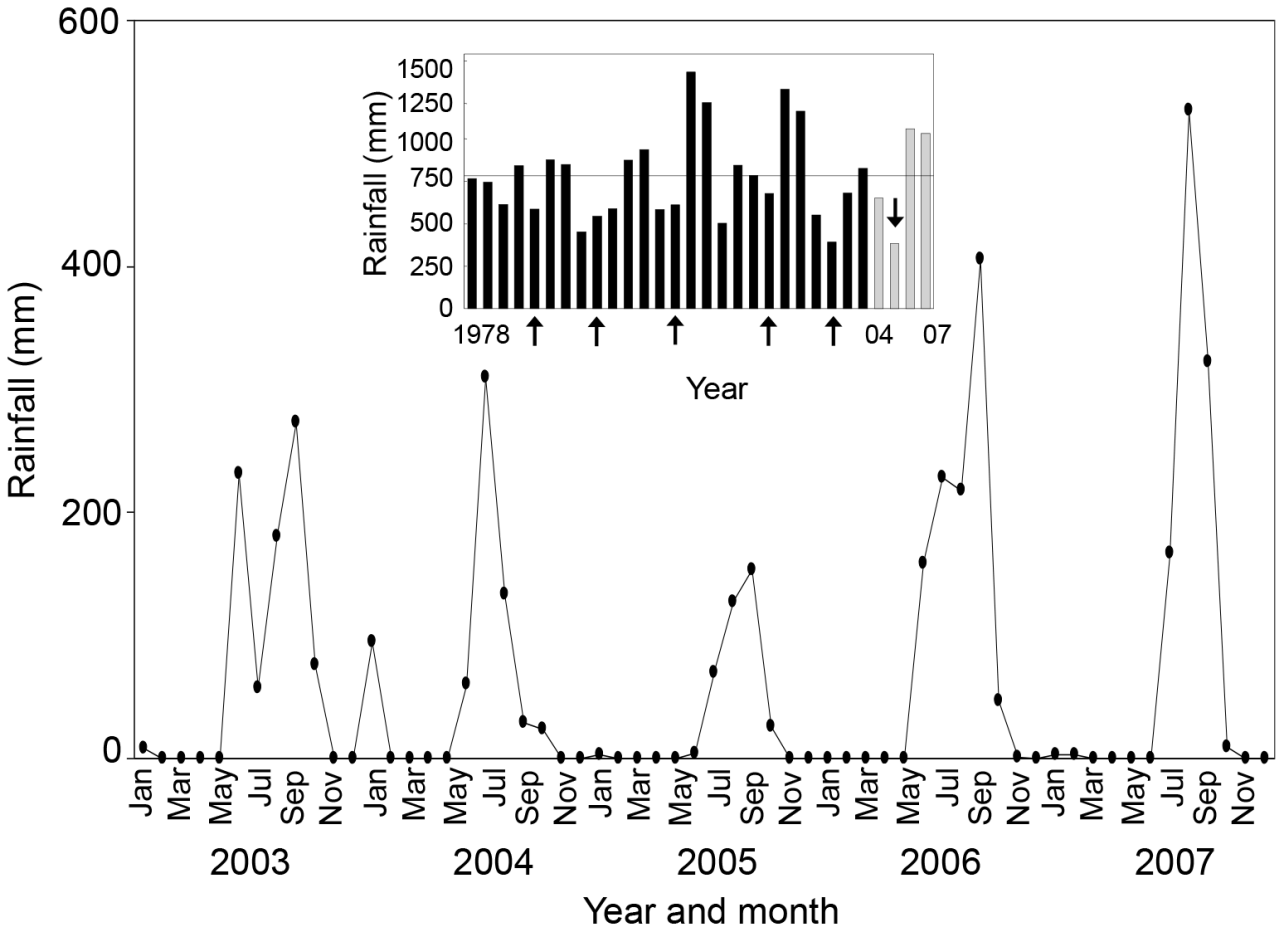
Long-term yearly and seasonal rainfall variation at Chamela, Western Mexico. Inset is shown annual rainfall records from1978 to 2007; grey bars correspond to the amount of rainfall recorded during the four years of the present study. Arrows point out El Niño Southern Oscillation (ENSO) years: 1982 (585.2 mm), 1986 (545.6 mm), 1991 (611 mm), 1997 (679 mm), 2001 (392 mm), and 2005 (384 mm). In the larger graph is shown monthly rainfall variation from 2003 to 2007. Note the strong drought episode caused by the ENSO event occurred in 2005, which produced the drier year in the last three decades before 2007.

The study was conducted at the Chamela-Cuixmala Biosphere Reserve (19°30′ N, 105°03′ W) and surrounding rural areas in the La Huerta municipality, Jalisco, Mexico. Mean annual temperature is 22.1°C and mean annual precipitation is 731 mm with wide inter-annual (384 to 1393 mm) and strong inter-seasonal variation (Figure 1). Almost 93% of the annual rainfall occurs from June to October, with a long dry season from November to the end of May. The main vegetation type is TDF. Canopy height varies between 5 and 10-m [42] and most trees and shrubs have small diameters at breast height (dbh <10-cm). During the dry season around 95% of all the plants drop their leaves. TDF covers about 30% of La Huerta municipality while cattle pasture fields and crops dominate the rest of the landscape and managed forests [43].

### Study System and Experimental Design

Nine abandoned cattle pasture sites, with fallow ages ranging from 0 to 12 years, and three conserved old-growth forest sites (OGF) were selected. The twelve sites were classified in four successional categories, each category with three replicates: i) Pasture (P; 0–1 years), ii) Early (E; 3–5 years), iii) Intermediate (I; 8–12 years), and iv) Old-Growth Forest (OGF). At each site, an area of 120×90-m (1.08 ha) was delimited with metal barbed wire posed (three parallel lines separated 50-cm each) to exclude cattle but not wild animals; in OGF only stakes were used to limit the study area. A permanent plot of 50×20-m was established at each site and 48 subplots (delimited in the corners with 0.5-m tall PVC poles) of 1-m^2^ each were homogenously established in each plot to include as much as possible the environmental heterogeneity. A total area of 576 m^2^ was sampled over the 12 sites; elsewhere [41] we provide details of the geographical location, biophysical and land use history characteristics of the study sites.

In October 2004, at the end of the rainy season, for each subplot, we tagged and measured every tree and shrub with heights between 10 and 100-cm (taken vertically from the ground level to the apical growth bud). Each recorded plant was measured in height and classified as a sprout, if it presented a physical connection with another plant, or as seedling, if cotyledons were present or if there was no evidence of it was re-sprouting from other plants. Taxonomic identification followed the nomenclature of Lott [44]; reference specimens were obtained outside of the plots and vouchers are available from the authors. Presence-absence data of species per successional category are shown for the whole study period (2004–2007) in the Supplementary Information (Table S1). Additional censuses were conducted over the following three years. In total, we did eight censuses, four at the end of the rainy seasons (October 2004, November 2005, November 2006, and October 2007), three at the initiation of the rainy season (September 2005, June 2006, and August 2007; in 2005 the rainy season began late; Figure 1) and one in the middle of the rainy season (September 2006). During these censuses new seedlings and sprouts reaching a height of 10-cm or more (hereafter referred as recruits) were recorded, measured and identified while surviving plants were re-measured for height.

Rainfall data used in this study comes from the Chamela Biological Field Station, Institute of Biology, National Autonomous University of Mexico [45]. All study sites are within a radius of 17-km from the Chamela Station.

### Data and Statistical Analysis

We calculated recruitment, growth, and mortality rates, as well as species gain and loss rates per successional category, separating dry seasons from the rainy ones, and for each studied year. These calculations were conducted considering the whole communities and separating trees from shrubs (i.e., by growth form), and seedlings from sprouts (i.e., by regeneration strategy). In sum, we monitored community dynamics over three one-year periods (which did not coincide with calendar years but for simplicity we will refer to them here after as years), encompassing three periods within dry seasons (October 2004 to September 2005, November 2005 to June 2006, and November 2006 to August 2007) and three within rainy seasons (September 2005 to November 2005, June 2006 to November 2006, and August 2007 to October 2007). It should be noted that duration of these periods (hereafter referred to as seasons) was variable. The length of the studied periods is different because the dry is longer than the rainy season. Moreover, the start and the length of each season varied among the studied years. Therefore, to have a standardized time scale of analysis, we calculated monthly rates per season. Recruitment rate (*RR*) was calculated as *RR* = [[(*n*+*r*)/*n*)]^30/t^]− 1, where *n* is the number of plants present at the beginning of the season, *r* is the number of new recruits recorded within a season and that survive until the end of that season, and *t* is the number of days elapsed between two censuses, made at the beginning and end of a given season. Mortality rate (*MR*) was calculated as *MR* = 1−[[1−(*m*/*n*)]^30/t^] where *m* is the number of initial plants that died from the beginning to the end of a given season. Growth rate (*GR*) was calculated as *GR* = (*h_f_ −h_i_*)/(*h_i_*)]*(30/*t*) where *h_i_* and *h_f_* are the plant height at the beginning and end of the season, respectively. Gain and loss rates of species were obtained using the same formulas for recruitment and mortality as mentioned before, but in these cases *n* was the number of species, *r* the number of new species, and *m* the number of species lost within a season. We are aware that different plant densities in ours plots could influence the estimated species loss and gain rates. Because most species had small sample sizes at the plot level, we could not do a rarefaction analysis to control for the effects of plant density. Instead, we used both plant density and species density as weighting factors to control for the effects of density, and test our hypothesis regarding community rates of change as described below. Both weighting factor gave similar results, and we therefore show only species density as a weighting factor in the results section.

Repeated measure analyses of variance (reANOVA) were used to evaluate the effects of calendar year (three levels: October 2004–November 2005, November 2005–November 2006, November 2006–October 2007), season (two levels: rainy and dry), and successional category (four levels: Pasture, Early, Intermediate, and Old-Growth Forest) on regenerative community rates. In addition, the analysis was redone using the factor regeneration strategy (two levels: seedlings and sprouts) or the factor growth form (two levels: trees and shrubs), separately. Because the initial plant density was different in all sites, reANOVA were weighted; for analyses of recruitment, mortality, and growth rates we used plant density per site (averaged over the three rainy season censuses) as the weighting factor while for gain and species loss analyses we used mean species density per site as a weighting factor. Table S2 in Supplementary Information shows the minimum and maximum values of plant density for each study site considering the whole study period (2004–2007). Both data for trees, shrubs, seedlings, sprouts, and the total community are shown. The interactions among factors are reported using the Wilk’s Lambda statistic. When necessary, response variables were transformed to meet the criteria of parametric analyses.

To remove the system intrinsic variation from that produced by the ENSO event, we carried out a detrending analysis, following Clark and Clark [46]. In this analysis, for each one of the twelve studied sites, we used as detrending procedure, where *x_i_* is the recorded rate value in a site for a given season and is the mean rate value over the six studied seasons for that site. This procedure provides the percentage of deviation of a rate recorded in a site at each season from the overall plot mean rate over all seasons. Because no direct effects of successional category on community rates were found (see results below), we used Friedman tests to assess only the effect of season on each of the detrended rates. Statistical analyses were performed using JMP 5.1., and SYSTAT 11, and untransformed data are shown in all graphs.

## Results

### Temporal Variation in Rainfall

During the study period, the first year (2005) received far less rainfall than the long-term mean annual rainfall recorded in our study locality (Figure 1). In that year occurred a severe ENSO event causing a very long dry season, resulting in the driest year of the previous three decades with climatological data (Figure 1). In the two previous years (2003 and 2004) annual rainfall was close to the long-term mean, and in the two years following to the ENSO year (2006 and 2007) annual rainfall was substantially above the long-term precipitation mean.

### Community Rates of Change Affecting Plant Density

#### Recruitment rate

**Figure 2:**
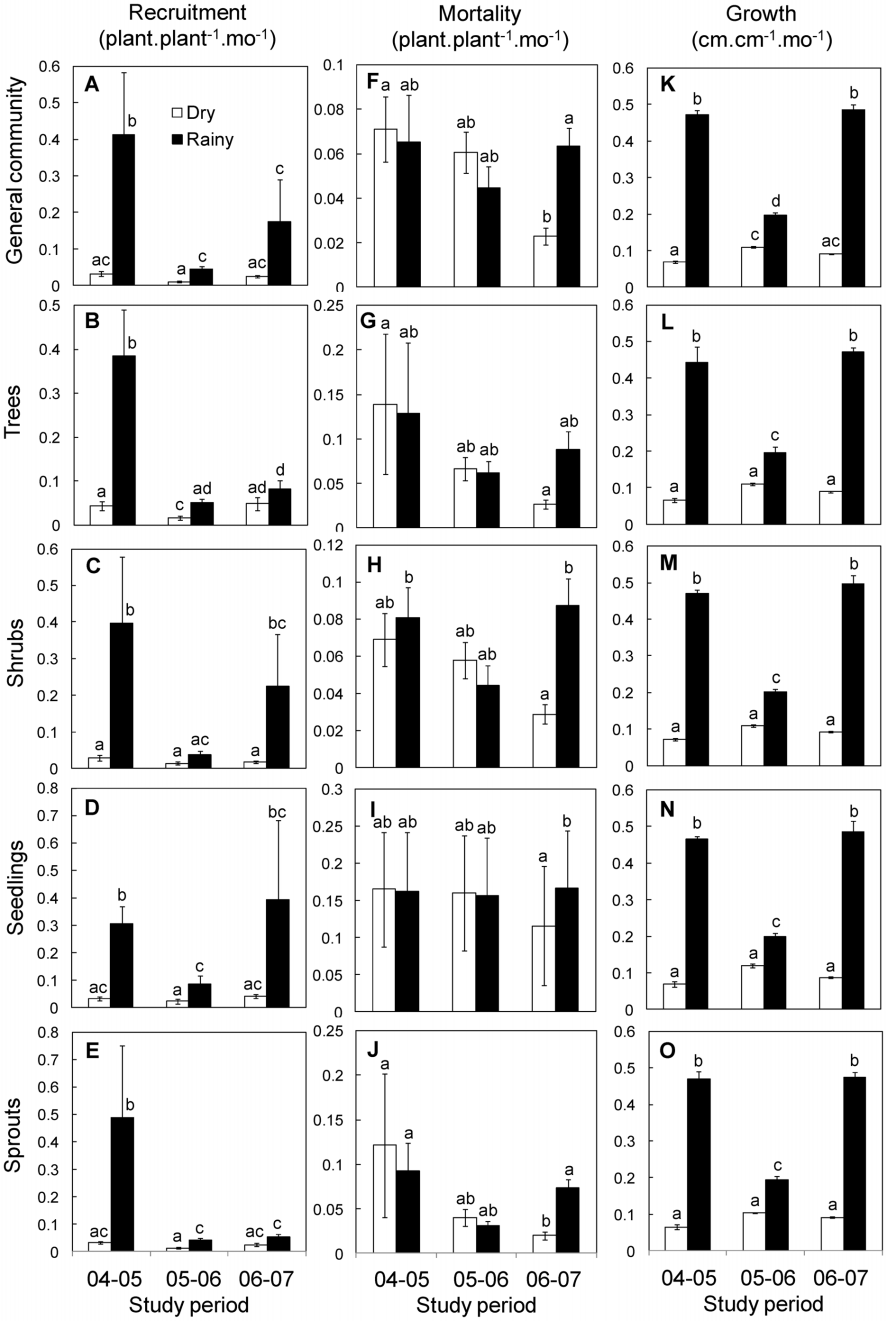
Seasonal variation of vital rates of regenerating communities over three studied years in successional TDF at Chamela, Mexico. Recruitment rate for A) whole community, B) trees, C) shrubs, D) seedlings, and E) sprouts communities; following the same order, mortality rates (F–J), and growth rates (K–O) are shown. Each graph shows mean monthly (±1 S.E, n = 12) community rates in the dry (open bars) and rainy season (black bars). Note the different scale of the y-axis in the different graphs.

At the whole community level, recruitment rate varied significantly among seasons, years and with the interaction between season and year (Table 1). Overall, recruitment in the rainy season was ten times higher (mean ± S.E = 0.21±0.07 plant.plant^−1^.mo^−1^) than in the dry season (0.02±0.00), and it was substantially higher in the ENSO year than in the following two years (Figure 2A). Even in the dry season recruitment decreased from the first (0.03±0.005) to the second year (0.01±0.002) and recovered a little bit in the third year (0.02±0.003). A detrended analysis confirmed these patterns (Figure S1A). Recruitment rate did not vary significantly with successional stage, probably because of the high inter-site variation. Although recruitment rate changed with the interaction between year and successional category (Table 1) such changes did not show any clear directional pattern.

**Table 1:** Results of repeated measure analysis of variance to assess the effects of year, season, successional category (SC) and interactions (×) among these factors on recruitment, mortality and growth rates of regenerative communities of tropical dry forest in Chamela, Mexico.

Recruitment rate											
		Whole community		Growth forms				Regeneration strategies			
				Trees		Shrubs		Seedlings		Sprouts	
Factors	*N*	*F*	*P*	*F*	*P*	*F*	*P*	*F*	*P*	*F*	*P*
**Year**	3	24.41	<0.0001	24.58	<0.001	9.56	0.001	9.39	0.001		NS
**Season**	2	88.23	<0.0001	62.45	<0.0001	62.5	<0.0001	87.83	<0.0001		NS
**SC**	4		NS		NS	4.24	0.02		NS		NS
**Year × Season**		10.3	0.001	11.46	0.0006	3.75	0.04	9.05	0.01		NS
**Year × SC**		3.14	0.01	3.51	0.007	3.33	0.01		NS		NS
**Mortality rate**											
**Year**	3	7.1	0.004		NS	9.21	0.001	9.7	0.001	6.84	0.006
**Year × Season**		15.81	<0.0001		NS	14.28	0.0001	3.84	0.04	22.27	0.0001
**Year × SC**			NS		NS		NS	4.95	0.001	3.0	0.02
**Growth rate**											
**Year**	3	322.1	<0.0001	283.3	<0.0001	422.7	<0.0001	425.7	<0.0001	494.0	<0.0001
**Season**	2	2179	<0.0001	1496	<0.0001	2048	<0.0001	1547	<0.0001	1633	<0.0001
**Year × Season**		447.8	<0.0001	358.4	<0.0001	562.9	<0.0001	673.4	<0.0001	549.5	<0.0001

≤0.05); no significant effects are indicated by NS. Only factors that had significant effects on at least one rate are shown. Figures correspond to sample size (N), F and significant P values (

For growth forms, overall, the recruitment rate of shrubs (0.12±0.04) was significantly higher than that of trees (0.10±0.02; F_1,46_ = 8.73, P = 0.004). The recruitment of trees and of shrubs varied significantly among years, seasons and the interaction between year and successional category (Table 1), paralleling the changes observed in the whole community (Figure 2B). A detrended analysis showed that the recovery of recruitment in the rainy season of the third year was greater for shrubs than for trees (Figure S1B, C). For shrubs a significant effect of successional category was found (Pasture, 0.29±0.6; Early, 0.09±0.02; Intermediate 0.05±0.02; Old-Growth Forest, 0.04±0.01).

With respect to regeneration strategies, recruitment rate of seedlings (0.15±0.05) was significantly higher than that of sprouts (0.11±0.05; F_1,46_ = 237.2 P ≤ 0.001). Seedling recruitment varied among years and seasons with the minima observed one year after the ENSO event (Table 1; Figure 2D). On average, seedling recruitment was significantly higher in the rainy (0.26±0.09) than in the dry season (0.03±0.004). While seedling recruitment in the dry season was similarly small along years, in the rainy season it strongly reduced after the ENSO year and recovered two years later, as indicated by the significant interaction between year and season (Table 1, Figure 2D). Finally, recruitment rate of sprouts did not vary significantly with any of the analysed factor (Figure 2E); however, the detrended analysis showed that recruitment of sprouts to undergo a similar seasonal variation than seedlings, except that no recovery was observed in the rainy season of the third year (Figure S1D, E).

#### Mortality rate

At the whole community level, mortality rate differed significantly affected by year and by the interaction between year and season (Table 1). On average, after a peak in mortality during the ENSO year, it decreased during the following two years. During the ENSO and following year, mortality did not differ between seasons but in the third year mortality was higher in the rainy than in the dry season (Figure 2F). The detrended analysis confirmed these patterns and identified a noticeable decrease in mortality during the rainy season of the second year but mainly in the dry season of the third year (Figure S1F). No effects were observed due to successional category.

Over years, seasons, and successional categories, trees (0.09±0.02 plant plant^−1^ mo^−1^) and shrubs (0.06±0.005) displayed similar mortality rate and it followed the temporal pattern as observed in the whole communities. Tree mortality did not vary among years, seasons or successional categories (Figure 2G); however, detrended analysis identified a notorious reduction of tree mortality in the dry season of the third year (Figure S1G). Shrub mortality showed the lowest rate one year after the ENSO event. Shrub mortality was higher in the rainy than in the dry season only in the last study year, as indicated by the significant interaction between year and season (Table 1, Figure 2H). These results were confirmed by the detrended analysis (Figure S1H).

Mortality rate of seedlings (0.15±0.03) was twice as high as that of sprouts (0.06±0.01; F_1,46_ = 5.33, P = 0.02). Seedling mortality changed significantly among years with the ENSO year showing the highest mortality rate. Seedling mortality showed also an interaction between year and season; while mortality was similar between dry and rainy seasons during the first two years, in the third year rainy showed higher mortality rate than dry season (Figure 2I). This change was evidenced by the detrended analysis (Figure S1I).

Mortality rate of sprouts differed significantly among years, between seasons depending on the year (as indicated by the interaction between year and season), and among successional categories depending on the year (as indicated by the interaction between year and successional category). The highest sprouts mortality occurred in the ENSO year. During this and the following year mortality was similar in the rainy and dry seasons but in the third year mortality peaked in the rainy season (Figure 2J). The detendred analysis emphasized a reduction in mortality during the rainy season of the second year and the dry season of the third year; also, it identified a peak in mortality during the rainy season of the third year (Figure S1J). Along the three study years sprouts mortality remained similar in the Early, Intermediate and OGF successional categories; in contrast, in the Pasture category mortality was high in the first year (0.27±0.15) and low in the two following years (0.05±0.02 and 0.06±0.02, respectively).

#### Growth rate

At the whole community level, growth rate changed significantly among years and between seasons but not among successional categories (Table 1). Overall, growth was four times higher in the rainy (0.38±0.02 cm.cm^−1^.mo^−1^) than in the dry season (0.09±0.003). In general, the lowest growth rate in the rainy season occurred one year after the ENSO year while growth in the dry season remained similarly low along the years, as indicated by the significant interaction between year and season (Figure 2K). The detrended analysis showed that growth rate was severely reduced in the rainy season after the ENSO year and recovered in the rainy season of two years later (Figure S1K).

Regarding growth forms, overall, growth differences between trees (0.23±0.02) and shrubs (0.24±0.02) were not significant. Growth rate of trees and of shrubs was significantly affected by the year, season and their interaction, and their temporal growth patterns paralleled those documented for the whole communities (Figure 2L, M; Figure S1L, M).

Considering regeneration strategies, difference in growth rate of seedlings (0.24±0.02) and sprouts (0.23±0.02) were not significant. Growth rate of seedlings and of sprouts showed similar temporal variation to that above described, changing significantly with year, season, and their interaction (Figure 2N, O; Figure S1N, O).

### Community Rates Affecting Species Density

**Figure 3:**
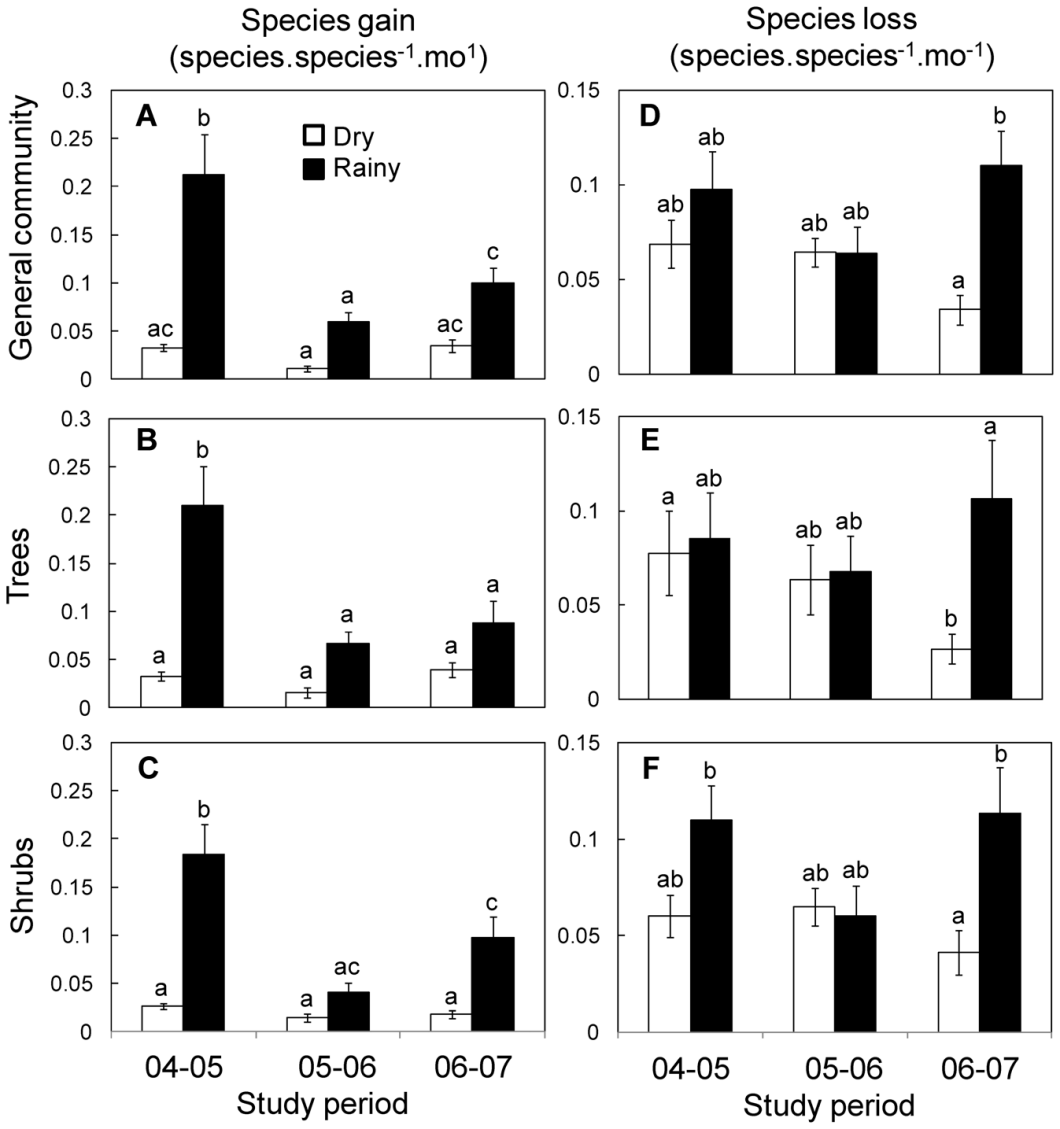
Seasonal variation of species gain and loss rates of regenerating communities over three studied years in successional TDF at Chamela, Mexico. Each graph shows mean monthly (±1 S.E, n = 12) community rates in the dry (open bars) and rainy season (black bars) over three one-year-periods. Species gain rate for A) whole community, B) trees, and C) shrubs; following the same order, species loss rates are shown in panels F to J. Note different scale of the y-axis in different graphs.

On average, species gain rate varied significantly among years, between seasons, and with their interaction (Table 2). Overall, species gain rate was four times higher in the rainy season (0.12±0.02 species.species^−1^.mo^−1^) than in the dry season (0.03±0.003). Among years, species gain was maximum in the ENSO year and minimum in the following year, especially in the rainy season (Figure 3A). Species gain rate was not significantly different between trees and shrubs. The temporal change in the species gain rate for trees and shrubs were generally the same as those observed in the whole communities (Figure 3B, C). The detrended analysis confirmed these results and identified a recovery of species gain in the rainy season two years after the ENSO event, particularly for shrubs (Figure S2A, B, C).

**Table 2:** Results of repeated measure analysis of variance to assess the effects of year, season, and interactions (×) among these factors on gain and loss species rates of regenerative communities of tropical dry forest in Chamela, Mexico.

Species gain rate							
		Whole community		Growth forms			
				Trees		Shrubs	
Factors	*N*	*F*	*P*	*F*	*P*	*F*	*P*
**Year**	3	14.85	<0.0001	12.96	0.0002	22.82	<0.0001
**Season**	2	78.37	<0.0001	54	<0.0001	62.3	<0.0001
**Year × Season**		11.18	0.0005	8.79	0.002	11.48	0.001
**Species loss rate**							
**Year**	3	3.65	0.04		NS		NS
**Season**	2	9.71	0.005		NS		NS
**Year × Season**		5.41	0.01	7.12	0.004	3.91	0.04

≤0.05); non-significant effects are indicated by NS. Only factors that had significant effects on at least one rate are shown. Figures correspond to sample size (N), F and significant P values (

At the whole community level, species loss rate varied significantly among years, between seasons, and with their interaction. On average, this rate was higher in the rainy season (0.10±0.01) compared with the dry season (0.05±0.01). Differences in species loss rate were not significant between seasons in the first and second study years but in the third year the loss of species was higher in the rainy than in the dry season (Figure 3D). The detrended analysis confirmed these results (Figure S2D).

Overall, species loss rate did not differ between trees and shrubs. This loss rate was only significantly affected by the interaction between year and season (Table 2). Differences in the loss of tree species between seasons were observed only in the third year when more species were lost in the rainy than in the dry season (Figure 3E). For shrubs species loss rate was higher in the rainy season in the first and third year of the study (Figure 3F) and these results were confirmed by the detrended analysis (Figure S2E, F).

### Temporal Trajectories in Plant and Species Density

**Figure 4:**
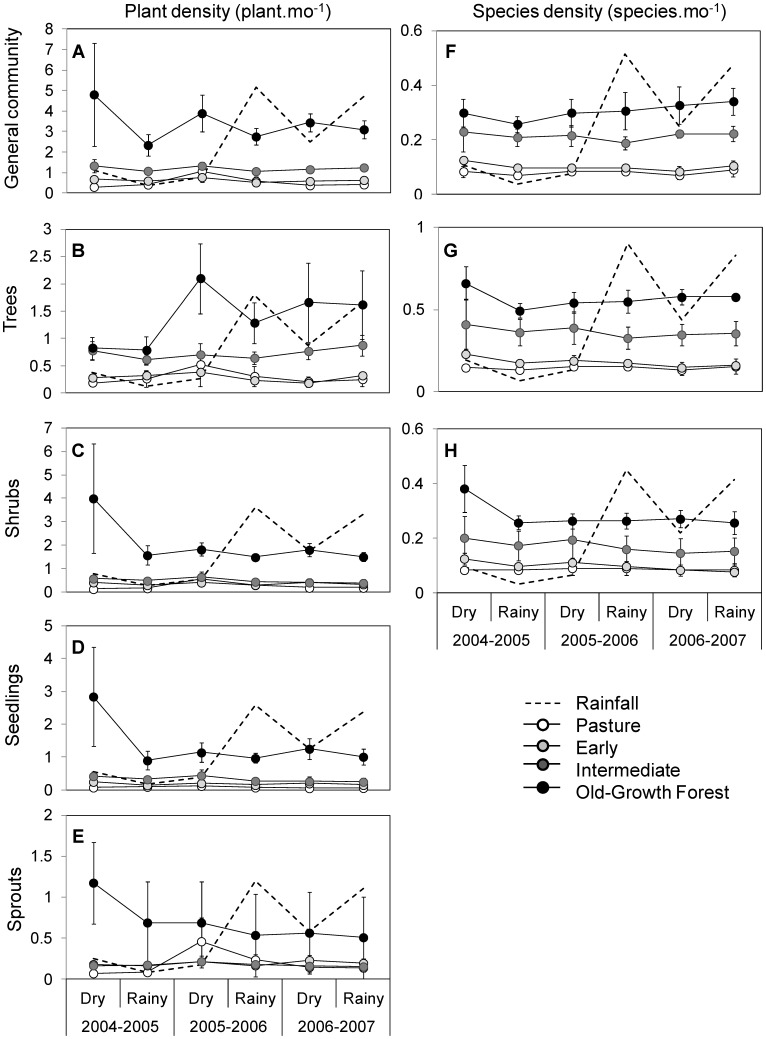
Yearly and seasonal changes in plant and species density of regenerative communities. Plant density for A) whole community, B) trees, C) shrubs, D) seedlings, and E) sprouts community. Species density for F) whole community, G) trees, and H) shrubs community. Each dot represents mean values (±1 S.E, n = 3). The dotted line indicates the monthly mean rainfall recorded at each season, scaled in agreement to each graph.

Figure 4 shows the temporal trajectories of plant density (Figure 4A–E) and species density (Figure 4F–H) along the four studied years for each successional category. The occurrence of ENSO in 2005 produced important changes both on plant density and species density, at the whole community level and separating trees from shrubs and seedlings from sprouts, especially in the old-growth forest. Such changes were smoothed out by the occurrence of two rainy years (2006 and 2007) subsequent to the ENSO year. In most successional categories, growth forms, and regeneration strategies, plant density and species density maintained similar values or decreased over time (Figure 4). However, there were some exceptions. Tree density increased over time in most successional categories but mainly in the Intermediate and OFG categories; as a result, differences in tree density among these successional categories and the younger ones became wider over time (Figure 4B). Stem density (but not species density) of shrubs increased over time in the Pasture but not in the others successional categories (Figure 4C), which resulted in a convergence of stem density of shrubs over time in the Pasture, Early and Intermediate categories (Figure 4C). Finally, sprout density increased with time in the Pasture, Early and Intermediate categories but decreased in the old-growth forest; as a result, after three years, differences in density of sprouts among successional categories reduced (Figure 4E).

## Discussion

Our study shows that temporal dynamics of TDF regenerative communities over old-field succession was governed by a strong inter-annual and inter-seasonal rainfall variation, which included an extraordinary ENSO event. Overall, the effects of temporal variation in rainfall did override those related with successional age, which indicate importance of global factors (ENSO, climatic regimes) over local (e.g. site abiotic factors related to fallow age) ones on the TDF regeneration dynamics in abandoned pasture fields.

### Temporal Variation in Rainfall and Community Dynamics

#### Recruitment, growth, and species gain

We hypothesized that recruitment, growth, and species gain rates would peak in the rainy season. This hypothesis was supported by our results. In the rainy season more water is available for the production of photosynthates used for biomass gain and reproduction [37], [38], [47]. The stem growth reductions during or after drought may also be due to shifts in carbohydrate allocation; at the beginning of the dry season, plants may invest in belowground root growth instead of stem growth, and in the wet season following drought plants may invest more in leaves, to recuperate photosynthetic carbon gain and replenish carbohydrate reserves [37], [38], [48], [49], [50]. In TDFs most wind-dispersed species produce seeds during the dry season that germinate during the following rainy season, while most animal dispersed species produce seeds during the rainy season that germinate either shortly afterwards or, in some cases, remain dormant until favourable growth conditions occur in future rainy seasons [16], [51], [52]. These kinds of reproductive behaviour of TDF plants may explain the higher recruitment and species gain rates of seedlings and sprouts observed during the rainy season in the three studied years.

The expectation that recruitment, growth and species gain rates would peak during the rainier years was not supported by our results. These rates were minimum in the rainy years of 2006 and 2007 and maximum in the dry ENSO year (2005). These unexpected results could be explained by storage of water and carbohydrate reserves, which are critical for future growth and sexual reproduction. In seasonal environments storage is amongst other factors, not only determined by current rainfall events but also by the amount of rain falling in previous years [35], [53], [54], [55], [56]. We believe that the severe drought in the ENSO year produced a strong shortage of stored reserves, which lead to reductions in recruitment, growth, and species gain rate of seedlings and sprouts observed after the ENSO year. Such long-lasting effect suggests that sexual activity and vegetative growth in TDF plants depend, amongst others, on reserves accumulated over periods longer than one year. Failure of seed production (due to scarcity of biotic pollination agents following a severe drought) could also play an important role in the reduction of recruitment and species gain after the ENSO event. Consistent with this idea, the peak of recruitment and high growth during the ENSO year (2005) could have been caused by sprouting, flowering, seeding activity and biomass gain stimulated by carbon gain during the previous three rainy years, which received more than two times the amount of rainfall recorded in the ENSO year (Figure 1). Also, it is possible that increment in recruitment and growth rates during the ENSO year reduced the stored reserves, limiting the next reproductive and growth events [37], [57]. Increased radiation in an ENSO year (caused by a reduced cloud cover and a reduced overstory), is also thought to contribute to the high recruitment, growth and species gain during the ENSO year. Yet, our results do not support this idea because canopy openness at 1 m above ground (%; mean ± S.E) did not show significant differences among the three studied years (F_2,33_ = 0.95, P = 0.4; 2005∶48.8±7.8; 2006∶45.8±9; and 2007∶32.4±8.3).

#### Mortality and species loss rates

Our hypothesis that mortality and species loss rates peak during the dry season and drier years was partially supported by our results. As expected, mortality and species losses peaked during the ENSO year but unexpectedly these rates were high not only in the dry but also in the rainy season of the ENSO year and the following year. This did happen even though the rainfall in the year following the ENSO event was 40% higher than the long-term mean annual precipitation recorded in Chamela (Figure 1). It is generally observed that drought leads to mortality, but the underlying mechanisms are still discussed [38]. Our results strongly suggest that severe and prolonged drought may not only lead to short-term mortality in the ENSO year, but also in the following years. Short-term drought-induced mortality can be attributed to cavitation and plant dessication [34], whereas reduced and/or depleted carbohydrate reserves may explain drought-induced mortality at the longer term (in the ENSO year and following years). A reduction of carbohydrate reserves may also weaken the plant against other stresses, such as pathogens and pests that may hit the plant in the following wet seasons [36], [58].

Another striking and unexpected result was that the mortality and species loss rates peaked in the rainy instead of the dry season after the two rainy years that followed the ENSO event. This result can be attributable to three possible causes. First, it is known that in rainy years TDF plants can store non-structural carbohydrates important for survival during drought spells and dry seasons [16], [18], [59]. Thus, we believe that the high precipitation that occurred in 2006 (Figure 1) enabled surviving plants to store reserves that were important to reduce mortality and species loss rates during the dry season of the following year. Second, we believe that in rainy years biotic factors become important agents of mortality for regenerative plants. Biotic damages caused by herbivores and pathogens have been shown to be important sources of mortality for seedlings and small plants in TDF during the rainy season [16], [60], [61], [62], [63]. Third, a depletion of carbohydrate reserves during prolonged droughts, may reduce the ability of plants to defend themselves, or recover from herbivores and pathogens [36], [38], [58], who show high densities during rainy seasons, increase. We do not believe that limitation in light resources *per se* was an important source of mortality in rainy years. In a previous study we showed that light availability in the understory is strongly reduced during the rainy season from Pasture to OGF successional categories [41]. However, our results show that mortality was independent of successional category at every studied year. The fact that ENSO had a similar impact on early and late successional communities suggest that pioneers (i.e., that are more abundant early in succession) and more late successional species (that are more abundant in the old-growth forest) respond in a similar way to drought. The ratio between evergreen and deciduous species may, however, shift after ENSO events; evergreen species are physiologically more drought tolerant than drought-avoiding deciduous species, so when ENSO hits during the wet season one might expect the evergreen species to survive better, leading a shift in community composition. It should further be investigated whether soil nutrient limitation, herbivores and pathogens present important mortality agents in the rainy season of rainy years.

### Effect of Growth Form and Regeneration Strategies on Regeneration Dynamics

The hypothesis that mortality of seedlings and trees is higher than that of shrubs and sprouts was partially supported by our results. Although seedlings suffered higher mortality than sprouts, trees showed similar mortality as shrubs. The small amounts of maternal resources (contained in the cotyledons), small sizes, limited root systems, and soft tissues of seedlings make them more susceptible to die due to biotic and abiotic damages [62], [64], [65], [66] than sprouts, which generally are bigger and receive higher amount of resources from the mother plant. The fragility of seedlings could be enhanced during severe droughts or heavy rains [24]. Our results show, however, that seedlings die at similar rates in both the dry and rainy season along the three years (Figure 2I). It is possible that mortality factors related to water limitation play an important role during the dry season while biotic agents of mortality become more important for seedlings during the rainy season.

### Effect of Successional Age on Regeneration Dynamics

We predicted that the effects of the temporal variation in rainfall would be higher at early than at late successional stages was not supported by our results. The only significant direct effect of successional category on the dynamics of regenerating communities was related to recruitment of shrubs. Such effects was related to the recruitment of *Mimosa* and *Senna* species in the Pasture successional category, which frequently act as pioneer plants in abandoned pastures and cornfields in Mesoamerican TDFs [67], [68], [69]. These species have high capacity to germinate (92–100%) and withstand the high temperatures and low soil water availability prevailing in open, disturbed sites [70].

The absence of clear effects of seasonality and successional category on community dynamics indicate that a strong drought year (ENSO) has an overwhelming effect on the regenerating plant community, swamping the more subtle effects of local factors. These findings suggest that severe drought episodes, like those caused by ENSO, may delay or slow down the regeneration rates of the TDF in abandoned agriculture fields, independently of successional age.

An increasing number of studies have pointed out the importance of ENSO events in tropical forests ecology. It has been documented ENSO effects on growth trajectories of pioneer trees in secondary TDF [71] and on tree demography [72], [73], reproductive behaviour of trees [74], [75], forest dynamics [76], biomass dynamics and carbon balance [77], [78], and successional dynamics [4] in other tropical forest biomes. The change in the rainfall pattern could produce disproportionate recruitment and mortality in different species; these variations could involve ecological, functional and ecosystems changes. Under current scenarios of global climate change, with ENSO events increasing in intensity and frequency in tropical regions, the dynamics of regenerative communities in seasonal areas could become even more complex.

## Conclusion

The dynamics of the studied TDF regenerative communities was strongly affected by temporal variability in rainfall but hardly by successional age. Regeneration dynamics did not only depend on the amount of rain falling in a given rainy season or year but also on previous rainfall events. Storage of non-structural carbohydrates for future survival, growth and reproduction, and biotic interactions like herbivory, pathogens, and biotic pollinators, acting in concert with temporal rainfall regimes, are some of the possible factors that may explain the observed dynamics during TDF old-field succession.

Our study shows that global factors like ENSO can play a paramount role in controlling the tempo of TDF regeneration in abandoned fields. With a predicted increase in the frequency and severity of drought, such as caused by ENSO events, long-term studies are urgently needed to understand the impact of global climate change on the successional process of secondary forests in tropical areas. This is particularly relevant as the conservation of biodiversity and ecosystem functions and services will depend on the resilience of tropical forests to withstand the disturbances caused by agriculture and global climate change.

## Supporting Information

Figure S1 **Seasonal variation of detrended rates of change (recruitment, mortality and growth rates) of TDF regenerating successional communities at Chamela, Mexico.** Recruitment rate for A) whole community B) trees, C) shrubs, D) seedlings, and E) sprouts. Mortality rates are shown in graphs F to J and growth rates in graphs K to O. Detendred values for each season (dry season: open bars, rainy season: black bars) are given as mean percentage deviation (±1 S.E, n = 12) from the site’s overall mean rate over the six studied seasons. Note the different scale of the y-axis in the different graphs. Study periods correspond to three calendar years (only the two final digits of each year are given). Letters show significant differences, Friedman test (P≤0.05).
(TIFF)

Figure S2 **Seasonal variation of detrendred rates of change (species gain and loss rates) of TDF regenerative communities at Chamela, Mexico.** Species gain rate for A) whole community, B) trees, and C) shrubs; species loss rate for D) whole community, E) trees, and F) shrubs. Detendred values for each season (dry season: open bars, rainy season: black bars) are given as mean percentage deviation (±1 S.E, n = 12) from the site’s overall mean rate over the six studied seasons. Note the different scale of the y-axis in the different graphs. Study periods correspond to three calendar years. Only the two final digits of each year are given). Letters show significant differences, Friedman test (P≤0.05).
(TIFF)

Table S1 **Species registered (X) of shrubs and three at the regenerative communities (10–100 cm tall) in pastures (P), early (E), and intermediate (I) successional sites, and old-growth forest (OGF) of tropical dry forest in Chamela, Mexico.**
(PDF)

Table S2 **Minimum and maximum number of plants recorded in each study site over the whole study period (October 2004–October 2007).**
(PDF)
