# Supplementary figures and images for: Effects of ENSO and Temporal Rainfall Variation on the Dynamics of Successional Communities in Old-Field Succession of a Tropical Dry Forest
Source: PLoS One. 2013 Dec 12;8(12):e82040. doi: 10.1371/journal.pone.0082040 (PMC3861369; doi:10.1371/journal.pone.0082040)

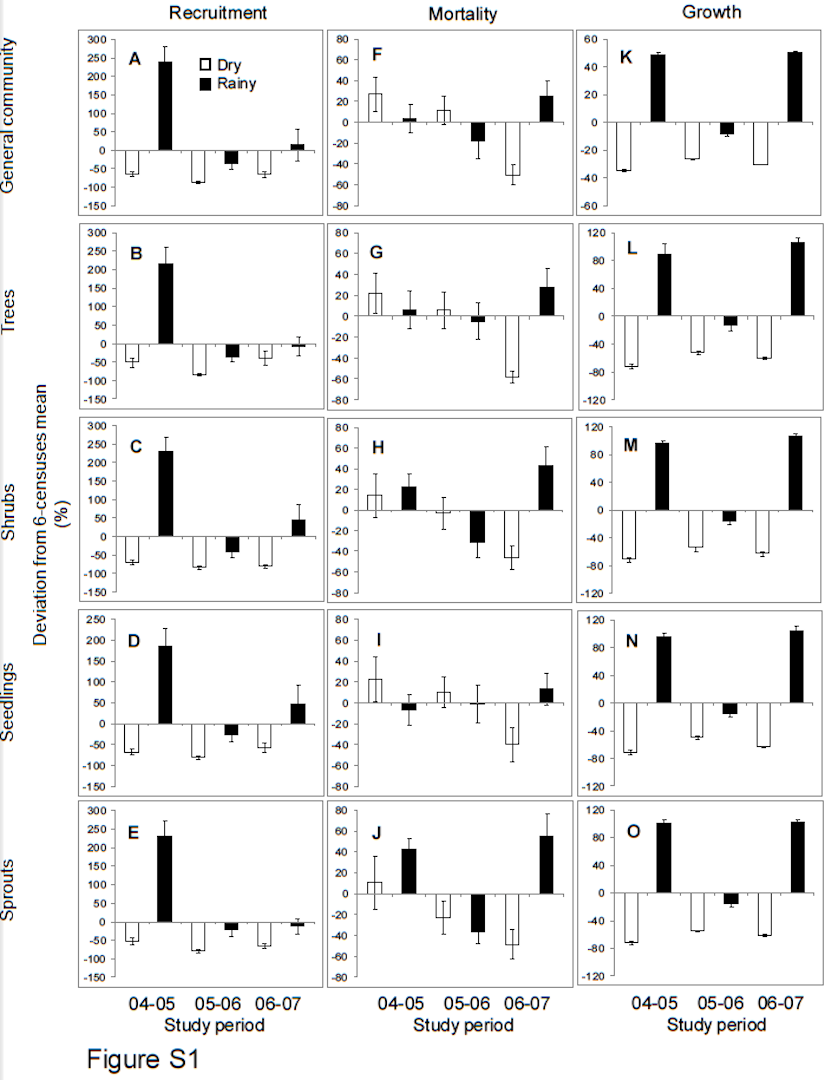

Supplement: Figure S1 — Seasonal variation of detrended rates of change (recruitment, mortality and growth rates) of TDF regenerating successional communities at Chamela, Mexico. Recruitment rate for A) whole community B) trees, C) shrubs, D) seedlings, and E) sprouts. Mortality rates are shown in graphs F to J and growth rates in graphs K to O. Detendred values for each season (dry season: open bars, rainy season: black bars) are given as mean percentage deviation (±1 S.E, n = 12) from the site’s overall mean rate over the six studied seasons. Note the different scale of the y-axis in the different graphs. Study periods correspond to three calendar years (only the two final digits of each year are given). Letters show significant differences, Friedman test (P≤0.05). (TIFF) [file pone.0082040.s001.tiff]

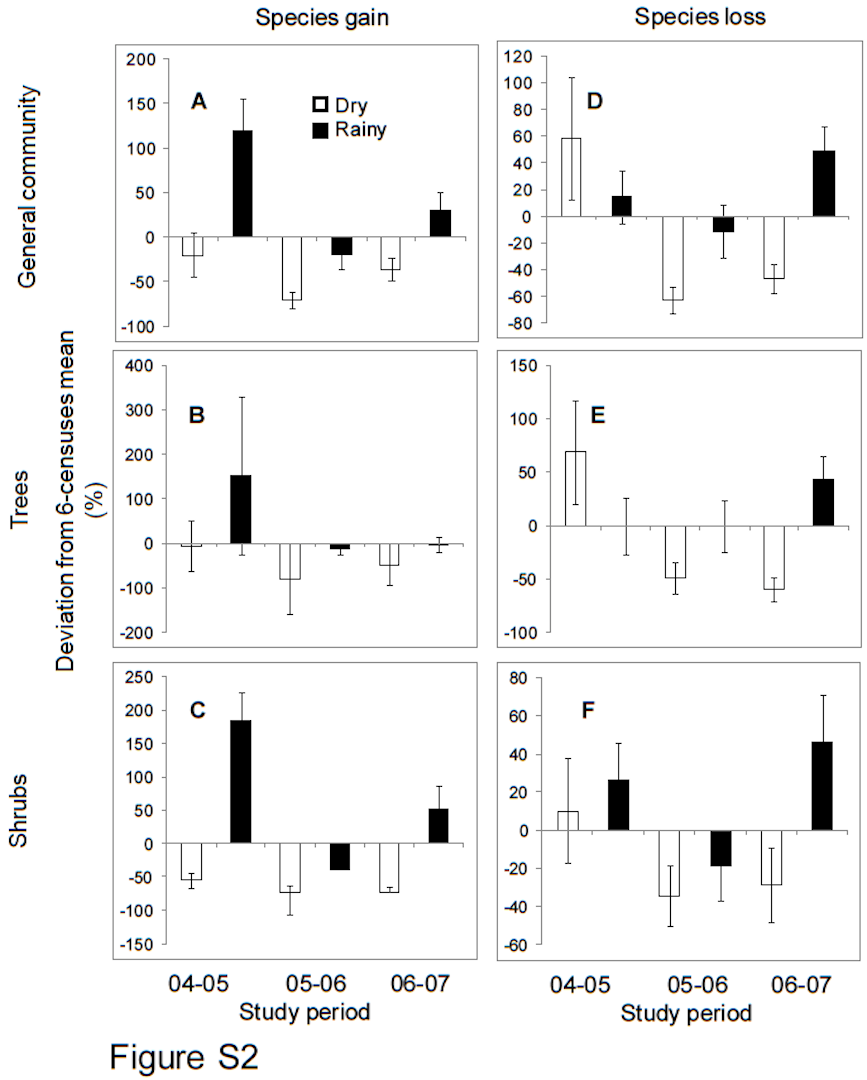

Supplement: Figure S2 — Seasonal variation of detrendred rates of change (species gain and loss rates) of TDF regenerative communities at Chamela, Mexico. Species gain rate for A) whole community, B) trees, and C) shrubs; species loss rate for D) whole community, E) trees, and F) shrubs. Detendred values for each season (dry season: open bars, rainy season: black bars) are given as mean percentage deviation (±1 S.E, n = 12) from the site’s overall mean rate over the six studied seasons. Note the different scale of the y-axis in the different graphs. Study periods correspond to three calendar years. Only the two final digits of each year are given). Letters show significant differences, Friedman test (P≤0.05). (TIFF) [file pone.0082040.s002.tiff]
