# Supplementary material for: Effects of ENSO and Temporal Rainfall Variation on the Dynamics of Successional Communities in Old-Field Succession of a Tropical Dry Forest
Source: PLoS One. 2013 Dec 12;8(12):e82040. doi: 10.1371/journal.pone.0082040 (PMC3861369; doi:10.1371/journal.pone.0082040)
Supplement: Table S1 — Species registered (X) of shrubs and three at the regenerative communities (10–100 cm tall) in pastures (P), early (E), and intermediate (I) successional sites, and old-growth forest (OGF) of tropical dry forest in Chamela, Mexico. (PDF) [file pone.0082040.s003.pdf]

Table S1. Species registered (X) of shrubs and three at the regenerative communities (10-100 cm tall) in pastures (P), early (E), and intermediate (I) successional sites, and old-growth forest (OGF) of tropical dry forest in Chamela, Mexico.

**Family**	**Species**	**Growth form**	**Successional category**			
			**P**	**E**	**I**	**OGF**
Acanthaceae	Justicia candicans (Nees) L. Benson.	shrub	X			X
	Justicia sp.	shrub				X
	Ruellia albiflora Fernald.	shrub				X
	Ruellia foetida Willd.	shrub		X	X	X
	Acanthaceaea sp.	shrub				X
Achatocarpaceae	Achatocarpus gracilis H. Walter	shrub			X	X
Anacardiaceae	Spondias purpurea L.	tree			X	X
Apocynaceae	Plumeria rubra L.	tree				X
	Rauvolfia tetraphylla L.	shrub	X	X	X	X
	Stemmadenia donell-smithii (Rose ex Donn.Sm.) Woods.	tree		X		
	Tabernaemontana amygdalifolia Jacq.	shrub		X		
Asteraceae	Asteraceae sp.	shrub				X
Bignoniaceae	Bignoniaceae sp.	shrub		X		
Bombacaeae	Ceiba aesculifolia (H.B.K.) Britt. & Baker.	tree			X	
Boraginaceae	Cordia alliodora (Ruiz & Pav.) Oken.	tree	X	X	X	X
Burseraceae	Bursera excelsa (Kunth) Engl.	tree				X
	Bursera instabilis McVaugh & Rzed.	tree				X
Capparaceae	Forchhammeria pallida Liebm.	tree			X	X
Convolvulaceae	Ipomea wolcottiana Rose.	tree				X
Ebenaceae	Diospyrus aequoris Standl.	shrub		X		
Ericaceae	Ericaceae sp.	shrub				X
Euphorbiaceae	Acalypha schiedeana Schltdl.	shrub			X	X
	Acalypha sp.	shrub		X		X
	Cnidoscolus spinosus Lundell.	tree	X		X	X
	Croton alamosanus Rose	shrub			X	X
	Croton pseudoniveus Lundell	tree			X	X
	Croton roxanae Croizat	tree		X	X	X
	Croton suberosus Kunth	shrub		X	X	X
	Croton sp.	shrub				X
	Jatropha malacophylla Standl.	tree		X	X	X
	Jatropha platyphylla Müll. Arg.	shrub				X

Family	Species	Growth form	Successional category			
			P	E	I	OGF
Euphorbiaceae	Pedilanthus calcaratus Schltdl.	tree			X	
	Phyllanthus botryanthus Müll. Arg.	shrub			X	X
	Phyllanthus mocinianus Baill.	tree	X		X	X
	Euphorbiaceae sp. 1	shrub			X	
	Euphorbiaceae sp. 2	shrub				X
Fabaceae	Acacia angustissima (Mill.) Kuntze	shrub			X	
	Acacia farnesiana (L.) Willd.	tree	X	X		X
	Acacia sp.	tree			X	
	Albizia tomentosa (Micheli) Standl.	tree			X	X
	Apoplanesia paniculata C. Presl	tree			X	X
	Bauhinia pauletia Pers.	shrub		X	X	X
	Caesalpinia coriaria (Jacq.) Willd.	tree			X	X
	Caesalpinia eriostachys Benth.	tree	X	X	X	X
	Caesalpinia platyloba S. Watson	tree			X	X
	Caesalpinia pulcherrima (L.) Sw.	shrub	X			X
	Caesalpinia sclerocarpa Standl.	tree	X	X	X	X
	Calliandra emarginata (Willd.) Benth.	shrub	X	X	X	X
	Calliandra formosa (Kunth) Benth.	shrub	X			
	Dalbergia congestiflora Pittier	tree				X
	Haematoxylum brasiletto H. Karst.	tree	X	X		
	Lonchocarpus constrictus Pitt.	tree	X	X	X	X
	Lonchocarpus eriocarinalis Micheli	tree	X			
	Lonchocarpus magallanesii M. Sousa	shrub	X			X
	Lysiloma microphyllum Benth.	tree			X	X
	Mimosa acantholoba (Humb. & Bonpl. ex Willd.) Poir.	tree	X			
	Mimosa arenosa (Willd.) Poir.	tree	X	X		
	Myrospermum frutescens Jacq.	shrub			X	
	Piptadenia constricta (Micheli & Rose ex Micheli) J.F. Macbr.	tree			X	X
	Piptadenia flava (Spreng. ex DC.) Benth.	tree		X		

Family	Species	Growth form	Successional category			
			P	E	I	OGF
Fabaceae	Pithecellobium unguis-cati (L.) Benth.	shrub				X
	Senna sp. 1	tree				X
	Senna sp. 2	shrub	X	X	X	X
	Zapoteca formosa (Kunth) H.M. Hern.	shrub	X		X	X
	Fabaceae sp. 1	shrub			X	X
	Fabaceae sp. 2	shrub				X
	Fabaceae sp. 3	shrub		X		
	Fabaceae sp. 4	tree			X	
	Fabaceae sp. 5	tree				X
	Fabaceae sp. 6	tree				X
	Fabaceae sp. 7	shrub			X	
	Fabaceae sp. 8	tree			X	
Flacourtiaceae	Casearia aculeata Jacq.	tree				X
	Casearia aff.arguta H.B.K.	shrub			X	
	Casearia corymbosa Kunth	shrub		X	X	
Hernandiaceae	Gyrocarpus jatrophifolius Domin	tree			X	X
Julianaceae	Amphyterigium adstringens (Schlecht.) Shiede.	tree				X
Malpighiaceae	Bunchosia palmeri S. Watson	shrub		X	X	
	Malpighiaceae sp.1	shrub				X
	Malpighiaceae sp.2	shrub	X			
Malvaceae	Malvaceae sp. 1	shrub				X
	Malvaceae sp. 2	shrub				X
Meliaceae	Trichilia havanensis Jacq.	shrub		X	X	
	Trichilia trifolia L.	tree				X
Moraceae	Brosimum alicastrum Sw.	tree				X
Myrtaceae	Psidium sartorianum (O. Berg) Nied.	tree				X
Nyctaginaceae	Guapira macrocarpa (Miranda) Miranda	tree	X	X	X	X
Polygonaceae	Coccoloba liebmanii Lindau	tree	X	X	X	X
	Ruprechtia fusca Fernald	tree				X
Rubiaceae	Exostema caribaeum (Jacq.) Roem. & Schult.	tree		X		
	Hintonia latiflora (Sessé & Moc. Ex DC.)	tree				X
	Randia aculeata L.	shrub		X		X
	Rubiaceae sp.	shrub			X	

Family	Species	Growth form	Successional category			
			P	E	I	OGF
Rutaceae	Amyris madrensis S. Watson	shrub				X
	Helietta lottiae F. Chiang	tree			X	
	Zanthoxylum fagara (L.) Sarg.	tree		X	X	X
Sapindaceae	Thouinia paucidentata Radlk.	tree				X
Sterculiaceae	Guazuma ulmifolia Lam.	tree				X
Theophrastaceae	Jacquinia pungens A. Gray	tree	X		X	
Tiliaceae	Heliocarpus pallidus Rose	tree		X	X	X
	Tiliaceae sp. 1	shrub				X
	Tiliaceae sp. 2	shrub				X
	Tiliaceae sp. 3	shrub				X
Ulmaceae	Phyllostylon rhamnoides (J. Poiss.) Taub.	tree			X	
Zygophyllaceae	Guaiacum coulteri A. Gray	tree			X	
	Zygophyllaceae sp.	shrub			X	
Morphospecies	Msp. 1	shrub				X
	Msp. 2	tree				X
	Msp. 3	shrub				X
	Msp. 4	tree			X	
	Msp. 5	shrub				X
	Msp. 6	shrub				X
	Msp. 7	shrub			X	
	Msp. 8	shrub		X		
	Msp. 9	shrub			X	
	Msp. 10	shrub				X
	Msp. 11	shrub	X			
	Msp. 12	shrub				X
	Msp. 13	shrub		X		
	Msp. 14	shrub				X
	Msp. 15	shrub				X
	Msp. 16	shrub				X
	Msp. 17	shrub				X
	Msp. 18	shrub				X
	Msp. 19	shrub			X	
	Msp. 20	shrub				X
	Msp. 21	tree				X
	Msp. 22	shrub				X
	Msp. 23	shrub			X	
	Msp. 24	shrub			X	
	Msp. 25	shrub				X
	Msp. 26	tree				X
