# Supplementary material for: Effects of ENSO and Temporal Rainfall Variation on the Dynamics of Successional Communities in Old-Field Succession of a Tropical Dry Forest
Source: PLoS One. 2013 Dec 12;8(12):e82040. doi: 10.1371/journal.pone.0082040 (PMC3861369; doi:10.1371/journal.pone.0082040)
Supplement: Table S2 — Minimum and maximum number of plants recorded in each study site over the whole study period (October 2004–October 2007). (PDF) [file pone.0082040.s004.pdf]

Table S2. Minimum and maximum number of plants recorded in each study site over the whole study period (October 2004 - October 2007).

Site	SC	Trees	Shrubs	Seedlings	Sprouts	Total
Ma1	Pasture	14-63	6-19	16-69	4-18	20-82
Cr0	Pasture	1-14	4-54	3-6	2-54	5-59
Za0	Pasture	5-8	2-26	1-20	6-11	9-31
Ca4	Early	12-23	9-18	0-7	23-30	23-36
Ra3	Early	10-17	8-15	4-17	10-20	18-29
Cr5	Early	3-25	20-36	21-45	10-21	31-59
Ca12	Intermediate	36-67	15-28	30-51	20-38	57-84
Cr12	Intermediate	22-50	29-54	36-75	13-24	51-88
Ra10	Intermediate	21-46	8-15	22-35	11-29	34-56
Gar	OGF	35-148	54-95	99-212	18-28	127-235
Tej1	OGF	27-121	66-415	48-322	52-150	127-472
Tej2	OGF	23-41	38-112	41-119	16-31	61-150

SC, Successional category. Minimum and maximum values do not always correspond to the beginning and final census of the study period.
